# Supplementary figures and images for: Mitochondrial complex I deficiency leads to the retardation of early embryonic development in Ndufs4 knockout mice
Source: PeerJ. 2017 May 18;5:e3339. doi: 10.7717/peerj.3339 (PMC5438584; doi:10.7717/peerj.3339)

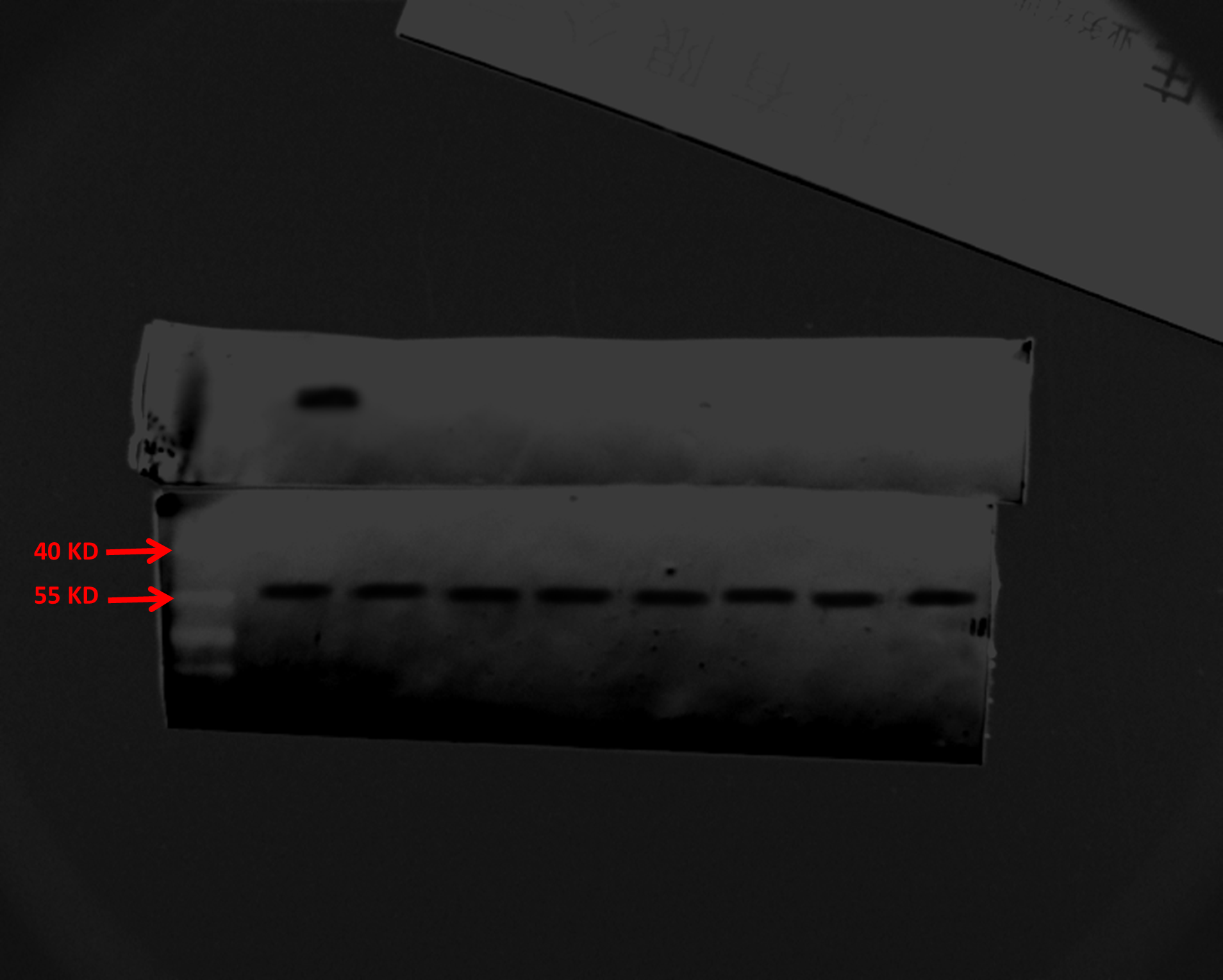

Supplement: Supplemental Information 1 — S1 showed the picture with labelled markers and NDUFS4 and ACTIN. S2 showed the picture of S1 at a lower exposure for distinct marker bands. S3 showed the raw data of S1. S4 showed the raw data of S2. [file peerj-05-3339-s001.zip › western blot/S1.tif]

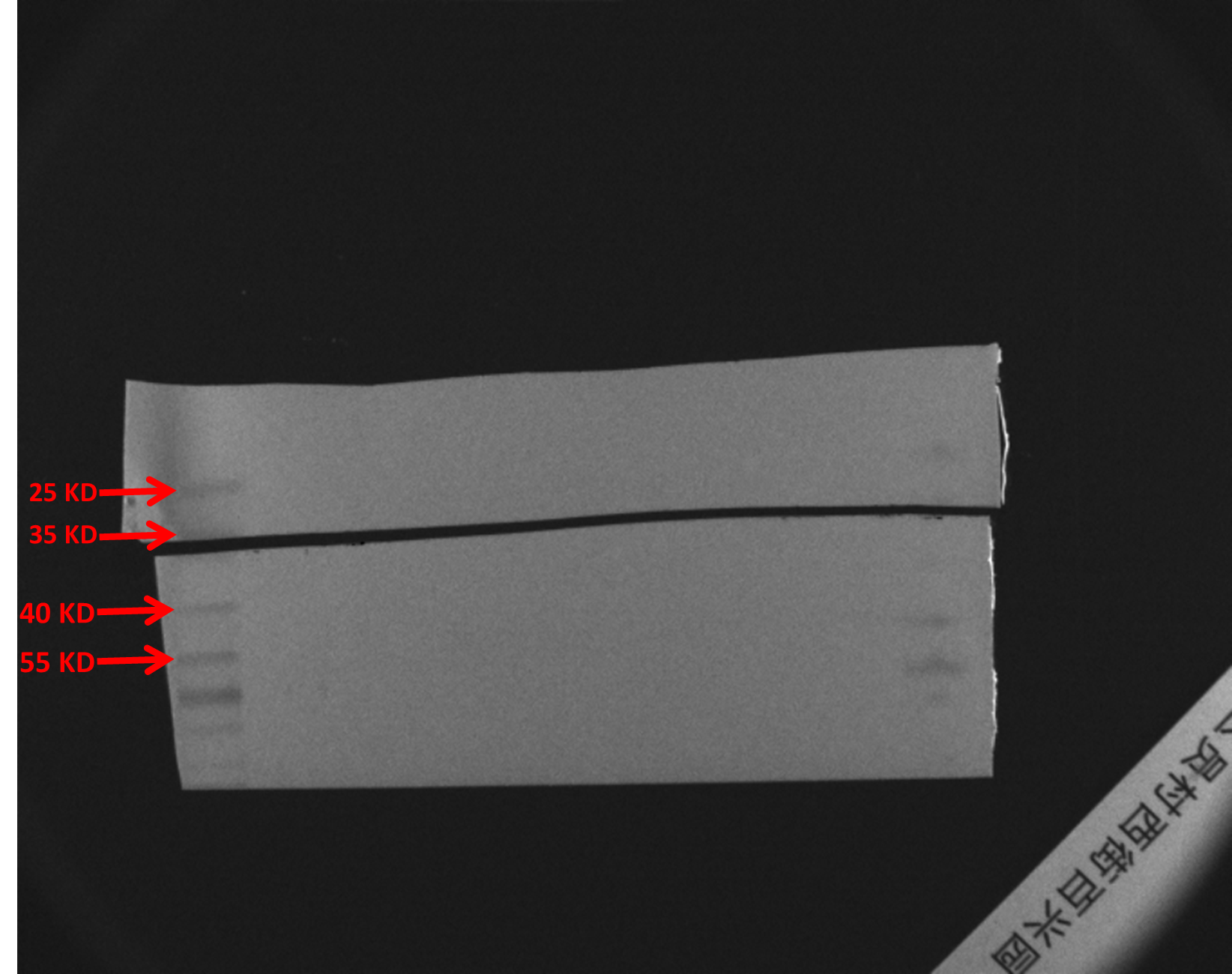

Supplement: Supplemental Information 1 — S1 showed the picture with labelled markers and NDUFS4 and ACTIN. S2 showed the picture of S1 at a lower exposure for distinct marker bands. S3 showed the raw data of S1. S4 showed the raw data of S2. [file peerj-05-3339-s001.zip › western blot/S2.tif]

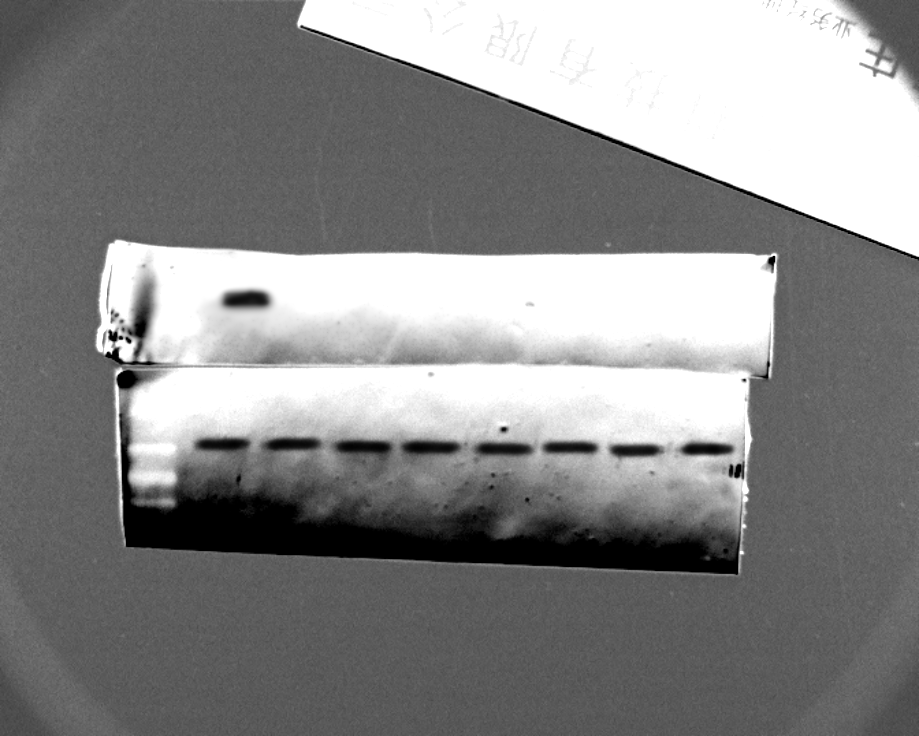

Supplement: Supplemental Information 1 — S1 showed the picture with labelled markers and NDUFS4 and ACTIN. S2 showed the picture of S1 at a lower exposure for distinct marker bands. S3 showed the raw data of S1. S4 showed the raw data of S2. [file peerj-05-3339-s001.zip › western blot/S3.tif]

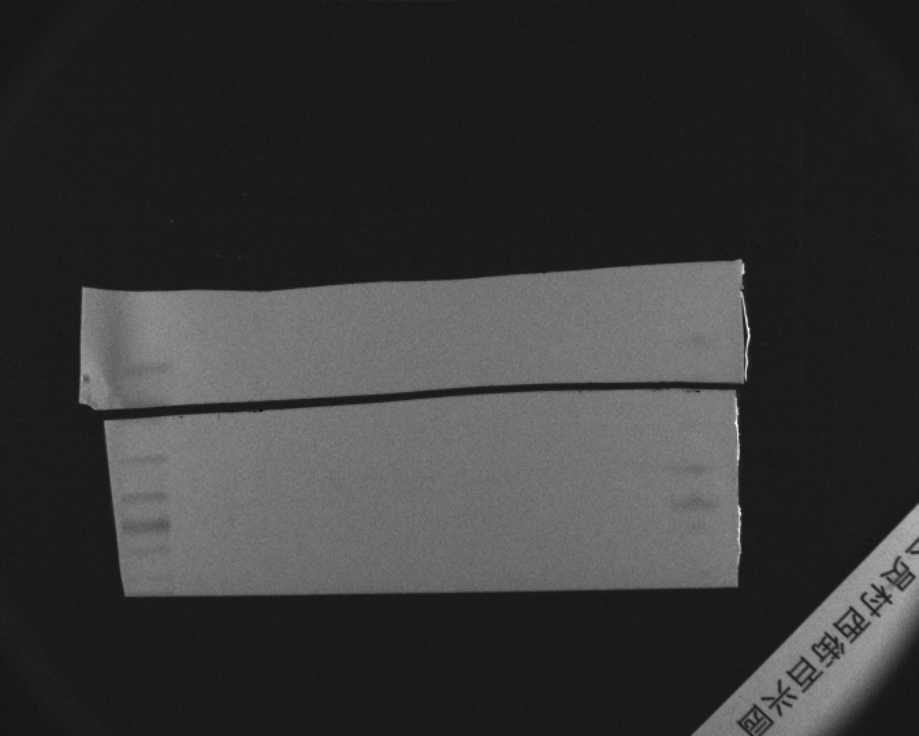

Supplement: Supplemental Information 1 — S1 showed the picture with labelled markers and NDUFS4 and ACTIN. S2 showed the picture of S1 at a lower exposure for distinct marker bands. S3 showed the raw data of S1. S4 showed the raw data of S2. [file peerj-05-3339-s001.zip › western blot/S4.tif]
