# Supplementary material for: Mitochondrial complex I deficiency leads to the retardation of early embryonic development in Ndufs4 knockout mice
Source: PeerJ. 2017 May 18;5:e3339. doi: 10.7717/peerj.3339 (PMC5438584; doi:10.7717/peerj.3339)
Supplement: Table S1 [file peerj-05-3339-s002.docx]

Table S1 Off-target analysis

| Site name | Gene name | Sequence | Indel mutation frequency  (Mutant/Total) | Coordinate | Detection Method |
| --- | --- | --- | --- | --- | --- |
| Target Ndufs4 | Ndufs4 | TATAACAGTTGATGAGAAAC**TAA** | / | chr13: 114351447 | Sanger sequencing |
| OT1 Ndufs4 | Helt | TCTATCACTTTATGAGAAAC**CGG** | None | chr8: 46293561 | Sanger sequencing |
| OT2 Ndufs4 | Cd33 | TCTAACAGTTAATGATAAAC**AGG** | None | chr7: 43531564 | Sanger sequencing |
| OT3 Ndufs4 | Gm37188 | GATAGCATTTGATGAGAATC**AGG** | None | chr9: 104303778 | Sanger sequencing |
| OT4 Ndufs4 | Gm2950 | CGTAACAGTTGATGAGAAAT**TGG** | None | chr9: 99220393 | Sanger sequencing |
| OT5 Ndufs4 | Cldn7 | TCTAACCGTTGATGGGAAAT**AGG** | None | chr11: 69965252 | Sanger sequencing |

OT, off-target; /, not tested. The other 14 predicted sites located in the noncoding sequences.
