# Supplementary material for: Mitochondrial complex I deficiency leads to the retardation of early embryonic development in Ndufs4 knockout mice
Source: PeerJ. 2017 May 18;5:e3339. doi: 10.7717/peerj.3339 (PMC5438584; doi:10.7717/peerj.3339)
Supplement: Table S2 [file peerj-05-3339-s003.docx]

Table S2 Primers used in the off-target analysis

| **Gene Target** | **Direction** **Primer (5’ to 3’)** | |
| --- | --- | --- |
|  | **F** | **R** |
| Helt | ATCTCCAGGATCTCCGCCTT | CTTGGCAGGGATGTGTAGGG |
| Cd33 | GAGGCTGGAGTGTGGCTTAG | GGTGGGGAGAGTTGTCATGG |
| Gm37188 | CCTTCCCCTTTCCTCCTCCT | CTAAGCCTCCAGCACTGGTC |
| Gm2950 | CCTGCAAGGACAATTGCATCT | GATGGCGGTGGTCTCAATGT |
| Cldn7 | CAGCACGGGTGATTTTACCG | TGACGTCACTGAAGGGACAC |
